# Supplementary material for: Experimental Yellow Fever in Squirrel Monkey: Characterization of Liver In Situ Immune Response
Source: Viruses. 2023 Feb 16;15(2):551. doi: 10.3390/v15020551 (PMC9961022; doi:10.3390/v15020551)
Supplement: Supplementary file 1 [file viruses-15-00551-s001.zip › viruses-1937085-supplementary.pdf]

viruses

MDPI

Supplementary Materials

Table S1. Relation of primary antibodies used in immunohistochemical reactions.

Cytokine	Brand/Code	pH	Dilution
S100	Dako/Z311	9.0	1:800
CD11b	ABCAM/ab52478	9.0	1:100
CD57	Labvision/MS-136	7.2	1:50
CD4	Novocastra/NCL-L-CD4-1F6	7.2	1:40
CD20	Dako/M755	7.2	1:300
Caspase 3	Abcam ab 13847	7.2	1:100
MLKL	Abcam ab 184718	7.2	1:100
iNOS	Abcam/NB300-605	6.0	1:100
Lisozima	Dako/A099	9.0	1:2000
IFN-γ	RD Systems/MAB 285	9.0	1:30
TNF-α	NOVUS/NBP1-19532	9.0	1:500
INF-β	Abcam 140211	6.0	1:100
IL-8	RD Systems/AF208NA	9.0	1:25
IL-17	Abcam ab79056	7.2	1:100
VCAM-1	RD Systems/BBA19	6.0	1:300
ICAM-1	Abcam ab	6.0	1:50
VLA-4	NB110-55525	6.0	1:100
IL-4	Abcam 9622	6.0	1:100
IL-10	RD Systems/AF217NA	9.0	1:20
IL-35	NOVUS/NBP2-27362	6.0	1:100
TGF-β	Santa Cruz/SC82	9.0	1:400

Table S2. Quantitative analysis of immunologically labeled cells.

Mean					Graduated Reticle (Area: 0.0625 mm²)					Standard Deviation				
S100					S100					S100				
DPI	Z3	Z2	Z1	PT	DPI	Z3	Z2	Z1	PT	DPI	Z3	Z2	Z1	PT
1	11.6	19.2	7.4	27.6	1	185.6	307.2	118.4	441.6	1	11.6	19.2	7.4	27.6
2	6.9	13	4.8	23.4	2	110.4	208	76.8	374.4	2	2.5	3.0	1.8	2.9
3	8.6	14.1	5.3	26.8	3	137.6	225.6	84.8	428.8	3	2.0	3.1	1.9	2.9
4	7.7	11.7	4.5	28.2	4	123.2	187.2	72	451.2	4	1.5	2.8	1.3	7.2
5	7.6	10.9	5.2	29.2	5	121.6	174.4	83.2	467.2	5	2.4	1.9	1.6	4.7
6	9.6	27.1	10	24	6	153.6	433.6	160	384	6	9.6	27.1	10.0	24.0
7	6.8	10.6	2.7	23.4	7	108.8	169.6	43.2	374.4	7	2.2	8.5	2.4	3.0
10	5.4	13.1	7.1	16.2	10	86.4	209.6	113.6	259.2	10	1.6	2.2	2.1	3.4
20	5.9	12.8	5.2	20.8	20	94.4	204.8	83.2	332.8	20	2.6	1.9	2.4	2.7
30	3.9	10	5.1	17.4	30	62.4	160	81.6	278.4	30	1.2	1.8	3.4	5.1
NC	1.5	4	0.2	1.5	NC	24	64	3.2	24	NC	2.2	1.6	2.0	3.8
CD11B					CD11B					CD11B				
DPI	Z3	Z2	Z1	PT	DPI	Z3	Z2	Z1	PT	DPI	Z3	Z2	Z1	PT
1	16.5	17.4	10.7	20.4	1	264	278.4	171.2	326.4	1	3.2	3.0	3.3	3.1
2	4.5	10.4	7.6	2	2	72	166.4	121.6	32	2	1.8	1.9	2.5	0.7
3	7.9	10.8	6.2	4	3	126.4	172.8	99.2	64	3	2.0	1.9	2.7	2.8
4	8.8	9.4	4.3	5	4	140.8	150.4	68.8	80	4	2.3	2.4	1.2	1.6
5	4.1	6.4	2.2	1.8	5	65.6	102.4	35.2	28.8	5	1.3	1.7	0.6	0.4
6	3.7	15.3	6.1	6.4	6	59.2	244.8	97.6	102.4	6	0.7	3.9	2.5	1.1
7	11.1	9.7	5.3	4.7	7	177.6	155.2	84.8	75.2	7	4.7	2.0	1.5	1.8
10	14.8	16	5.8	4.8	10	236.8	256	92.8	76.8	10	2.9	4.0	1.8	1.5
20	7	8.6	4.9	4.2	20	112	137.6	78.4	67.2	20	1.2	2.2	1.3	2.2
30	10.8	14.2	7.7	4.4	30	172.8	227.2	123.2	70.4	30	3.0	2.1	2.5	1.1
NC	1	1.4	6.3	1.1	NC	16	22.4	100.8	17.6	NC	0	0	0	0
CD57					CD57					CD57				
DPI	Z3	Z2	Z1	PT	DPI	Z3	Z2	Z1	PT	DPI	Z3	Z2	Z1	PT

1	12.1	17.4	5.3	8	1	193.6	278.4	84.8	128	1	1.1	2.1	2.1	2.0
2	7.2	12.3	2.8	1.8	2	115.2	196.8	44.8	28.8	2	2.6	1.9	1.2	0.8
3	8	11.9	2.7	5	3	128	190.4	43.2	80	3	2.0	1.7	0.8	2.0
4	7.5	11.5	2	4.2	4	120	184	32	67.2	4	2.5	1.6	1.4	2.9
5	6.3	13	4.6	3.6	5	100.8	208	73.6	57.6	5	1.5	1.3	1.3	1.1
6	4.7	19.8	6.4	7.8	6	75.2	316.8	102.4	124.8	6	1.4	5.0	1.8	1.5
7	5.8	10.9	2.6	6.4	7	92.8	174.4	41.6	102.4	7	1.9	2.1	0.8	4.2
10	2.3	4.5	0.6	0.4	10	36.8	72	9.6	6.4	10	1.9	1.2	1.0	0.9
20	5.4	9	0.9	5.2	20	86.4	144	14.4	83.2	20	2.2	1.4	0.9	1.1
30	6.5	10.3	4.8	4.6	30	104	164.8	76.8	73.6	30	1.4	3.7	1.3	5.1
NC	0.3	0.8	0.4	0.2	NC	4.8	12.8	6.4	3.2	NC	0	0	0	0

		**CD4**					**CD4**					**CD4**		
**DPI**	**Z3**	**Z2**	**Z1**	**PT**	**DPI**	**Z3**	**Z2**	**Z1**	**PT**	**DPI**	**Z3**	**Z2**	**Z1**	**PT**
1	14.7	20.8	7.4	4.2	1	235.2	332.8	118.4	67.2	1	2.5	3.5	2.4	0.8
2	5.5	12.4	2.4	0.6	2	88	198.4	38.4	9.6	2	3.0	3.1	1.2	0.9
3	6.4	10.9	0.7	0	3	102.4	174.4	11.2	0	3	1.6	2.1	1.2	0.0
4	6.5	9.2	0.4	0.2	4	104	147.2	6.4	3.2	4	2.4	1.4	0.7	0.4
5	4.9	8.8	0.7	0.2	5	78.4	140.8	11.2	3.2	5	1.1	2.7	1.3	0.4
6	9	15	7	11	6	144	240	112	176	6	0.0	0.0	0.0	0.0
7	2.7	5.8	1.9	0.2	7	43.2	92.8	30.4	3.2	7	2.2	2.1	1.3	0.4
10	0.1	1.9	0.1	0.2	10	1.6	30.4	1.6	3.2	10	0.3	2.8	0.3	0.4
20	1.6	3.3	0	0	20	25.6	52.8	0	0	20	1.4	2.6	0.0	0.0
30	4.4	9.4	2.3	0	30	70.4	150.4	36.8	0	30	1.5	4.2	1.2	0.0
NC	0.8	1.2	0.3	0.3	NC	12.8	19.2	4.8	4.8	NC	0	0	0	0

		**CD-20**					**CD-20**					**CD-20**		
**DPI**	**Z3**	**Z2**	**Z1**	**PT**	**DPI**	**Z3**	**Z2**	**Z1**	**PT**	**DPI**	**Z3**	**Z2**	**Z1**	**PT**
1	10.2	11.8	4.6	4.2	1	163.2	188.8	73.6	67.2	1	2.0	3.7	1.9	1.1
2	8.3	11.6	2.7	1.8	2	132.8	185.6	43.2	28.8	2	2.2	2.2	1.9	1.1
3	7.8	11.8	3	0	3	124.8	188.8	48	0	3	3.3	2.5	2.3	0.0
4	8.5	11.1	0.8	3	4	136	177.6	12.8	48	4	1.5	1.9	1.2	1.6
5	7.3	11.6	2	0	5	116.8	185.6	32	0	5	1.8	2.2	1.3	0.0
6	0	0	0	0	6	0	0	0	0	6	0.0	0.0	0.0	0.0
7	3.8	7	0	1.2	7	60.8	112	0	19.2	7	1.7	1.6	0.0	0.8
10	1.5	5.1	0.3	0.6	10	24	81.6	4.8	9.6	10	1.6	1.7	0.7	0.9
20	6.6	8.5	1.6	0	20	105.6	136	25.6	0	20	1.8	1.3	1.5	0.0
30	5.7	11.7	5.4	0.4	30	91.2	187.2	86.4	6.4	30	1.1	2.1	1.6	0.5
NC	0.6	0.4	0.2	0.3	NC	9.6	6.4	3.2	4.8	NC	0	0	0	0

		**VCAM- 1**					**VCAM- 1**					**VCAM- 1**		
**DPI**	**Z3**	**Z2**	**Z1**	**PT**	**DPI**	**Z3**	**Z2**	**Z1**	**PT**	**DPI**	**Z3**	**Z2**	**Z1**	**PT**
1	10.5	14.8	2.7	11.8	1	168	236.8	43.2	188.8	1	1.8	2.2	1.6	2.3
2	8.1	13.4	3.9	1.8	2	129.6	214.4	62.4	28.8	2	1.4	2.2	1.3	0.8
3	7.6	15	4.2	16.8	3	121.6	240	67.2	268.8	3	1.6	2.3	1.8	4.9
4	8.3	12.2	4.8	11.8	4	132.8	195.2	76.8	188.8	4	2.2	2.3	1.5	2.3
5	9.5	16.6	7.5	9.2	5	152	265.6	120	147.2	5	2.5	2.5	1.2	2.9
6	0	9	4.6	14.6	6	0	144	73.6	233.6	6	0.0	1.8	3.2	3.5
7	5.9	11.8	4	18.4	7	94.4	188.8	64	294.4	7	1.4	2.4	1.1	3.0
10	2.4	6.6	1.2	11.2	10	38.4	105.6	19.2	179.2	10	1.9	1.1	0.8	1.3
20	2.1	5.6	3.7	13.4	20	33.6	89.6	59.2	214.4	20	1.2	1.4	1.3	5.8
30	7.3	13.3	4.7	9.6	30	116.8	212.8	75.2	153.6	30	1.5	1.4	1.3	3.3
NC	1.3	1.9	0.8	1.8	NC	20.8	30.4	12.8	28.8	NC	0	0	0	0

		**ICAM-1**					**ICAM-1**					**ICAM-1**		
**DPI**	**Z3**	**Z2**	**Z1**	**PT**	**DPI**	**Z3**	**Z2**	**Z1**	**PT**	**DPI**	**Z3**	**Z2**	**Z1**	**PT**
1	6.1	11.1	3.3	4.6	1	97.6	177.6	52.8	73.6	1	1.6	1.8	1.3	0.9
2	7.2	10.6	3.3	3	2	115.2	169.6	52.8	48	2	2.7	1.7	1.1	1.2
3	7.3	11.3	3.2	13.4	3	116.8	180.8	51.2	214.4	3	1.1	1.3	0.8	2.7
4	7.5	12.6	4.4	13.2	4	120	201.6	70.4	211.2	4	1.4	3.0	1.7	3.0
5	8.3	15.7	6.6	21	5	132.8	251.2	105.6	336	5	1.9	1.9	1.6	4.4

6	5.7	11.9	6.2	17	6	91.2	190.4	99.2	272	6	2.7	2.8	2.3	7.6
7	8.1	17.2	8.1	7.4	7	129.6	275.2	129.6	118.4	7	2.8	3.0	2.3	1.7
10	2.3	4.6	1.1	2.4	10	36.8	73.6	17.6	38.4	10	1.9	1.6	1.3	0.5
20	6.7	8.5	4.3	3.8	20	107.2	136	68.8	60.8	20	1.6	2.3	1.5	1.1
30	5.1	9.5	3.6	6.3	30	81.6	152	57.6	100.8	30	0	0	0	0
NC	1.1	1.6	1.2	1.7	NC	17.6	25.6	19.2	27.2	NC	0	0	0	0

		VLA-4					VLA-4					VLA-4		
**DPI**	**Z3**	**Z2**	**Z1**	**PT**	**DPI**	**Z3**	**Z2**	**Z1**	**PT**	**DPI**	**Z3**	**Z2**	**Z1**	**PT**
1	7.4	7.9	1.6	3.2	1	118.4	126.4	25.6	51.2	1	1.6	1.0	2.1	1.1
2	6	8.9	1.7	2.8	2	96	142.4	27.2	44.8	2	2.0	1.9	1.8	0.8
3	7	10	1.8	3.2	3	112	160	28.8	51.2	3	1.2	1.6	1.4	1.1
4	6.8	8.4	2.8	5.8	4	108.8	134.4	44.8	92.8	4	1.5	2.2	2.8	2.2
5	6.8	7.7	3.4	4.8	5	108.8	123.2	54.4	76.8	5	1.5	2.2	2.7	2.0
6	7.5	9.3	2.5	5	6	120	148.8	40	80	6	1.1	1.3	1.1	1.9
7	6.2	8.2	2.6	2.7	7	99.2	131.2	41.6	42.7	7	1.9	1.6	1.3	0.8
10	6.7	7.3	2.4	3	10	107.2	116.8	38.4	48	10	1.3	1.3	1.4	1.2
20	6.1	7.2	2.2	2.4	20	97.6	115.2	35.2	38.4	20	0.7	1.8	0.8	1.1
30	6.3	7.9	1.8	2.8	30	100.8	126.4	28.8	44.8	30	1.3	1.0	1.2	1.3
NC	0.9	1.3	0.9	1.6	NC	14.4	20.8	14.4	25.6	NC	0	0	0	0

		Caspase 3					Caspase 3					Caspase 3		
**DPI**	**Z3**	**Z2**	**Z1**	**PT**	**DPI**	**Z3**	**Z2**	**Z1**	**PT**	**DPI**	**Z3**	**Z2**	**Z1**	**PT**
1	8.6	10.6	4	5.6	1	137.6	169.6	64	89.6	1	1.51	1.65	1.76	1.67
2	7.3	11.5	5.8	7.2	2	116.8	184	92.8	115.2	2	1.83	2.12	2.44	2.49
3	11.5	17.8	8.8	11.8	3	184	284.8	140.8	188.8	3	2.55	3.16	2.39	4.60
4	11.1	16.4	10.4	12.6	4	177.6	262.4	166.4	201.6	4	1.73	3.27	2.67	2.07
5	10.8	17.5	10.7	9.8	5	172.8	280	171.2	156.8	5	1.40	2.80	1.42	2.17
6	9.8	15.1	8	9	6	156.8	241.6	128	144	6	3.26	2.02	2.31	1.41
7	7.4	12.1	6.5	17.0	7	118.4	193.6	104	272.0	7	1.84	2.60	1.27	4.90
10	9.3	13	4.4	6	10	148.8	208	70.4	96	10	2.58	2.58	1.65	1.41
20	12.4	13.9	7	5.8	20	198.4	222.4	112	92.8	20	1.84	2.28	3.37	2.28
30	9.2	15.5	5.9	7	30	147.2	248	94.4	112	30	2.39	2.55	1.79	1.58
NC	1.3	0.9	1.6		NC	20.8	14.4	25.6	0	NC	0	0	0	0

		MLKL					MLKL					MLKL		
**DPI**	**Z3**	**Z2**	**Z1**	**PT**	**DPI**	**Z3**	**Z2**	**Z1**	**PT**	**DPI**	**Z3**	**Z2**	**Z1**	**PT**
1	5.2	8	2.5	4	1	83.2	128	40	64	1	1.2	2.0	1.0	1.2
2	2.1	2.9	1.5	5	2	33.6	46.4	24	80	2	1.0	1.6	0.8	2.4
3	2.8	7.4	2.5	4	3	44.8	118.4	40	64	3	0.9	3.1	1.1	4.5
4	7.3	8.8	4.1	20.8	4	116.8	140.8	65.6	332.8	4	2.2	1.8	1.1	4.3
5	3.8	5.6	1.9	2.6	5	60.8	89.6	30.4	41.6	5	1.2	1.8	1.7	2.4
6	12.6	19.3	13.5	15.5	6	201.6	308.8	216	248	6	1.6	3.3	2.5	4.2
7	2.1	3.5	2.2	5.2	7	33.6	56	35.2	83.2	7	1.0	1.3	1.4	2.3
10	1.3	2.5	0.6	6.4	10	20.8	40	9.6	102.4	10	0.5	1.0	0.7	2.4
20	2	6.8	2.4	6.6	20	32	108.8	38.4	105.6	20	0.7	1.9	1.5	0.9
30	2.2	5.8	5.6	9.6	30	35.2	92.8	89.6	153.6	30	1.0	1.2	1.5	3.1
NC	0.3	0.4	0.1	0	NC	4.8	6.4	1.6	0	NC	0	0	0	0

		INOS					INOS					INOS		
**DPI**	**Z3**	**Z2**	**Z1**	**PT**	**DPI**	**Z3**	**Z2**	**Z1**	**PT**	**DPI**	**Z3**	**Z2**	**Z1**	**PT**
1	10.7	14.9	8.3	7.6	1	171.2	238.4	132.8	121.6	1	2.5	3.1	1.7	1.8
2	10.9	14.6	8.5	8.6	2	174.4	233.6	136	137.6	2	1.8	2.7	1.1	1.9
3	11.4	15.4	9.1	8.8	3	182.4	246.4	145.6	140.8	3	2.1	2.6	1.1	1.5
4	11.4	14.7	9.6	7.8	4	182.4	235.2	153.6	124.8	4	2.7	2.4	2.0	0.8
5	12.2	18.6	11.3	8.6	5	195.2	297.6	180.8	137.6	5	2.1	3.2	2.1	0.9
6	14.1	21.8	15.2	14.2	6	225.6	348.8	243.2	227.2	6	2.4	3.7	3.3	2.9
7	11.2	13.4	10.3	8.6	7	179.2	214.4	164.8	137.6	7	1.4	2.8	1.9	1.8
10	10.1	12	9	9	10	161.6	192	144	144	10	1.7	2.1	1.5	2.1
20	9.1	10.2	8.3	7	20	145.6	163.2	132.8	112	20	1.2	1.6	1.5	2.1
30	10.2	10.1	7.3	5.8	30	163.2	161.6	116.8	92.8	30	2.0	1.8	1.8	1.3

NC	2	4	2	1.2	NC	32	64	32	19.2	NC	0	0	0	0

		**Lysozima**					**Lysozima**					**Lysozima**		
**DPI**	**Z3**	**Z2**	**Z1**	**PT**	**DPI**	**Z3**	**Z2**	**Z1**	**PT**	**DPI**	**Z3**	**Z2**	**Z1**	**PT**
1	15.6	18	5.6	20.4	1	249.6	288	89.6	326.4	1	2.9	2.7	1.6	2.4
2	10.2	13.5	3.8	9	2	163.2	216	60.8	144	2	1.6	3.3	1.0	0.5
3	10.7	10.9	5.8	24.6	3	171.2	174.4	92.8	393.6	3	2.4	2.4	2.1	8.0
4	16.1	13.5	6.1	20.8	4	257.6	216	97.6	332.8	4	4.7	3.1	2.0	6.2
5	16.4	16.8	9.4	10	5	262.4	268.8	150.4	160	5	3.6	2.2	4.4	1.5
6	11	16.3	8.1	10.1	6	176	260.8	129.6	161.6	6	2.9	3.3	2.8	1.5
7	17.4	15.5	7.5	21.2	7	278.4	248	120	339.2	7	4.7	3.7	2.1	2.9
10	10.7	13.4	5.1	10.2	10	171.2	214.4	81.6	163.2	10	2.8	3.3	1.2	3.6
20	6	8.3	4.2	5.8	20	96	132.8	67.2	92.8	20	3.2	3.3	1.5	1.8
30	6.6	10.7	4.9	8	30	105.6	171.2	78.4	128	30	1.8	2.9	1.2	1.2
NC	1.5	1.7	1.1	0.6	NC	24	27.2	17.6	9.6	NC	0	0	0	0

		**IFN- ϒ**					**IFN- ϒ**					**IFN- ϒ**		
**DPI**	**Z3**	**Z2**	**Z1**	**PT**	**DPI**	**Z3**	**Z2**	**Z1**	**PT**	**DPI**	**Z3**	**Z2**	**Z1**	**PT**
1	9.7	12	4.3	5.8	1	155.2	192	68.8	92.8	1	1.7	1.8	1.1	2.2
2	5.2	10.8	2.1	1.8	2	83.2	172.8	33.6	28.8	2	1.4	1.3	1.3	0.4
3	6.2	11.2	3.4	18.4	3	99.2	179.2	54.4	294.4	3	2.0	1.9	1.0	4.1
4	7.1	11.9	3.2	8.8	4	113.6	190.4	51.2	140.8	4	1.8	1.7	1.2	2.5
5	5.2	9.1	1.6	7.6	5	83.2	145.6	25.6	121.6	5	1.3	1.4	1.5	3.6
6	7.3	11.2	6	9.8	6	116.8	179.2	96	156.8	6	2.9	3.0	1.2	2.8
7	5	10.2	4.6	9.4	7	80	163.2	73.6	150.4	7	1.8	2.4	1.6	2.5
10	3.2	7.9	4.7	2.4	10	51.2	126.4	75.2	38.4	10	1.8	1.2	1.7	2.1
20	6.1	9.2	3.7	4.2	20	97.6	147.2	59.2	67.2	20	2.8	1.5	2.1	1.9
30	5	9.9	4	3	30	80	158.4	64	48	30	0.7	2.0	1.9	0.7
NC	2.7	3.1	0.8	1.2	NC	43.2	49.6	12.8	19.2	NC	0.0	0.0	0.0	0.0

		**IFN-β**					**IFN-β**					**IFN-β**		
**DPI**	**Z3**	**Z2**	**Z1**	**PT**	**DPI**	**Z3**	**Z2**	**Z1**	**PT**	**DPI**	**Z3**	**Z2**	**Z1**	**PT**
1	7.3	11	7.2	4.8	1	116.8	176	115.2	76.8	1	2.3	1.8	1.0	0.4
2	8.5	11.7	6.5	5.8	2	136	187.2	104	92.8	2	2.4	1.9	2.3	2.2
3	8.7	11.6	6.5	4.8	3	139.2	185.6	104	76.8	3	1.9	1.2	1.6	2.0
4	10.3	11.1	7.6	5	4	164.8	177.6	121.6	80	4	1.8	1.2	0.8	2.7
5	11.8	14.4	8.7	6.2	5	188.8	230.4	139.2	99.2	5	1.5	1.1	0.7	1.6
6	12.4	17.1	11.4	8.4	6	198.4	273.6	182.4	134.4	6	1.8	2.6	1.4	0.5
7	11.6	12.6	9	4.8	7	185.6	201.6	144	76.8	7	2.5	2.6	1.1	2.0
10	9.2	10.1	8.8	4.6	10	147.2	161.6	140.8	73.6	10	2.4	1.9	0.8	0.9
20	8.8	10.2	8.7	5.2	20	140.8	163.2	139.2	83.2	20	1.5	0.8	1.9	1.8
30	7.9	10.2	7.4	5.2	30	126.4	163.2	118.4	83.2	30	1.0	1.2	1.2	1.5
NC	2.3	2.8	1.1	1.9	NC	36.8	44.8	17.6	30.4	NC	0	0	0	0

		**TNF - α**					**TNF - α**					**TNF - α**		
**DPI**	**Z3**	**Z2**	**Z1**	**PT**	**DPI**	**Z3**	**Z2**	**Z1**	**PT**	**DPI**	**Z3**	**Z2**	**Z1**	**PT**
1	11.9	17.6	5.3	10.8	1	190.4	281.6	84.8	172.8	1	2.1	3.5	1.3	2.9
2	9.3	10.7	3.5	2.6	2	148.8	171.2	56	41.6	2	2.5	1.4	1.3	1.5
3	10.5	12.4	3.5	18.6	3	168	198.4	56	297.6	3	1.6	1.5	0.5	5.8
4	8.3	13.1	3.6	12	4	132.8	209.6	57.6	192	4	1.5	2.5	1.5	1.9
5	8.3	13.9	3	7.4	5	132.8	222.4	48	118.4	5	1.9	4.2	1.1	2.6
6	9.9	18.1	8.2	15	6	158.4	289.6	131.2	240	6	2.1	4.6	1.8	1.9
7	8.1	10.8	3	13.8	7	129.6	172.8	48	220.8	7	1.4	1.5	1.1	3.7
10	6.6	10.7	3.2	9.4	10	105.6	171.2	51.2	150.4	10	1.3	2.7	1.2	1.7
20	5.5	8.9	2.4	4.2	20	88	142.4	38.4	67.2	20	1.5	2.2	0.7	1.3
30	7	12.2	4.7	3.8	30	112	195.2	75.2	60.8	30	1.8	1.6	1.1	0.8
NC	3.1	4.2	1.1	1.9	NC	49.6	67.2	17.6	30.4	NC	0.0	0.0	0.0	0.0

		**IL- 8**					**IL- 8**					**IL- 8**		
**DPI**	**Z3**	**Z2**	**Z1**	**PT**	**DPI**	**Z3**	**Z2**	**Z1**	**PT**	**DPI**	**Z3**	**Z2**	**Z1**	**PT**

1	14	22	7	5	1	224	352	112	80	1	1.2	1.8	1	1.3
2	8	17	3	2	2	128	272	48	32	2	1.3	1.6	1.3	1.9
3	6	14	3	2	3	96	224	48	32	3	0.8	1.1	1.2	1.5
4	6	10	2	2.4	4	96	160	32	38.4	4	0.0	0.0	0.0	2.3
5	5.2	9.2	1	0	5	83.2	147.2	16	0	5	1.4	1.6	0.9	0.0
6	3	9.2	3.9	2	6	48	147.2	62.4	32	6	1.3	3.2	1.4	3.1
7	1.4	4.9	1.6	1.4	7	22.4	78.4	25.6	22.4	7	1.3	1.4	0.6	1.1
10	1.4	2.7	1.3	1.1	10	22.4	43.2	20.8	17.6	10	0.7	1.1	0.0	0.0
20	5.7	6.2	1.5	1	20	91.2	99.2	24	16	20	1.9	1.5	0.7	1.0
30	5.7	11.7	3.6	2.4	30	91.2	187.2	57.6	38.4	30	1.7	1.6	1.3	1.3
NC	4.2	5.3	1.3	2.4	NC	67.2	84.8	20.8	38.4	NC				

		**IL-17**					**IL-17**					**IL-17**		
**DPI**	**Z3**	**Z2**	**Z1**	**PT**	**DPI**	**Z3**	**Z2**	**Z1**	**PT**	**DPI**	**Z3**	**Z2**	**Z1**	**PT**
1	10	12	9	8	1	160	192	144	128	1	1.4	1.5	0.9	1.8
2	11	12	7	7	2	176	192	112	112	2	0.9	1.6	1.3	0.9
3	13	15	9	8	3	208	240	144	128	3	1.5	1.9	1.5	1.7
4	9	16	7	7	4	144	256	112	112	4	1.7	2	1.8	1.8
5	11	18	9	6	5	176	288	144	96	5	1.6	1.9	0.9	1
6	16	19	13	10	6	256	304	208	160	6	1.9	1.8	1	0.9
7	11	13	9	6.0	7	176	208	144	96.0	7	1	1.9	1.7	0.8
10	9	11	8	7	10	144	176	128	112	10	0.9	1.5	1.8	1.6
20	9	10	7	7	20	144	160	112	112	20	0.6	1.6	1.9	1.7
30	8	11	8	6	30	128	176	128	96	30	0.4	1.3	1.6	1.8
NC	0.9	1.3	0.9	1.6	NC	14.4	20.8	14.4	25.6	NC	0	0	0	0

		**IL-4**					**IL-4**					**IL-4**		
**DPI**	**Z3**	**Z2**	**Z1**	**PT**	**DPI**	**Z3**	**Z2**	**Z1**	**PT**	**DPI**	**Z3**	**Z2**	**Z1**	**PT**
1	10.1	11.9	8.1	6.8	1	161.6	190.4	129.6	108.8	1	1.9	1.1	1.4	1.1
2	11.3	13.2	7.9	7.4	2	180.8	211.2	126.4	118.4	2	1.3	1.6	1.5	1.5
3	11.1	14.2	8.8	7.8	3	177.6	227.2	140.8	124.8	3	1.9	2.6	1.5	0.8
4	11.2	13.8	8.1	7.6	4	179.2	220.8	129.6	121.6	4	1.9	1.9	1.2	1.5
5	12.4	18.3	10	6.4	5	198.4	292.8	160	102.4	5	1.7	1.4	1.2	1.3
6	11.7	20.4	8.9	8.4	6	187.2	326.4	142.4	134.4	6	2.6	2.1	1.4	0.9
7	9.9	11.8	8.7	5.6	7	158.4	188.8	139.2	89.6	7	1.7	2.2	1.3	0.9
10	8.2	11.3	8.1	5.6	10	131.2	180.8	129.6	89.6	10	0.9	3.0	1.2	1.9
20	8.6	11.4	8.1	6.4	20	137.6	182.4	129.6	102.4	20	1.1	2.3	1.0	1.3
30	8.9	11.4	8.4	5.6	30	142.4	182.4	134.4	89.6	30	0.9	2.1	1.3	1.3
NC	3	4.1	1.1	2.1	NC	48	65.6	17.6	33.6	NC	0	0	0	0

		**IL-10**					**IL-10**					**IL-10**		
**DPI**	**Z3**	**Z2**	**Z1**	**PT**	**DPI**	**Z3**	**Z2**	**Z1**	**PT**	**DPI**	**Z3**	**Z2**	**Z1**	**PT**
1	8.4	15.5	4.7	3.2	1	134.4	248	75.2	51.2	1	3.0	3.1	1.6	1.1
2	6.2	12.6	3.3	3.6	2	99.2	201.6	52.8	57.6	2	1.9	2.1	0.9	1.3
3	9.4	13.5	3.5	1.3	3	150.4	216	56	20.8	3	2.5	3.4	1.6	0.9
4	6.3	10.5	2.5	1.8	4	100.8	168	40	28.8	4	1.3	1.4	1.9	1.3
5	6.6	10	4	2.2	5	105.6	160	64	35.2	5	1.8	1.9	1.6	1.3
6	4.9	9.7	5.3	3.8	6	78.4	155.2	84.8	60.8	6	1.9	2.8	1.3	1.9
7	3	9	1.8	3.2	7	48	144	28.8	51.2	7	1.8	3.2	1.6	1.6
10	1.4	2.3	1.5	0.2	10	22.4	36.8	24	3.2	10	1.3	1.2	0.0	0.4
20	3.7	6.7	1.3	1.4	20	59.2	107.2	20.8	22.4	20	2.2	2.1	0.4	0.9
30	4.5	11.5	4.5	2.4	30	72	184	72	38.4	30	1.7	2.9	1.6	2.6
NC	2.1	3.6	1.5	3.1	NC	33.6	57.6	24	49.6	NC	0	0	0	

		**IL-35**					**IL-35**					**IL-35**		
**DPI**	**Z3**	**Z2**	**Z1**	**PT**	**DPI**	**Z3**	**Z2**	**Z1**	**PT**	**DPI**	**Z3**	**Z2**	**Z1**	**PT**
1	8.8	11.6	9.1	6.2	1	140.8	185.6	145.6	99.2	1	1.6	1.2	1.5	1.6
2	10	11.7	8.7	5.8	2	160	187.2	139.2	92.8	2	1.7	0.9	1.6	0.8
3	10	12	8.4	7	3	160	192	134.4	112	3	1.8	1.4	1.9	2.1
4	11.1	12	8.7	6.8	4	177.6	192	138.7	108.8	4	1.4	2.1	1.3	1.1
5	12	13.5	9.6	7.4	5	192	216	153.6	118.4	5	2.3	2.5	1.1	2.5

6	12	16.9	10	8.2	6	192	270.4	160	131.2	6	1.5	1.3	1.3	1.9
7	9.5	10.9	7.4	4.8	7	152	174.4	118.4	76.8	7	0.8	1.6	1.1	1.1
10	8.4	9.3	6.8	5.2	10	134.4	148.8	108.8	83.2	10	0.8	1.3	1.0	2.3
20	8.2	9.9	6.5	4.8	20	131.2	158.4	104	76.8	20	1.5	0.9	1.6	2.3
30	7.8	9.9	7	4.2	30	124.8	158.4	112	67.2	30	0.9	2.0	1.8	1.1
NC	2.1	3.3	1.5	2.2	NC	33.6	52.8	24	35.2	NC	0	0	0	0

		**TGF- β**					**TGF- β**					**TGF- β**		
**DPI**	**Z3**	**Z2**	**Z1**	**PT**	**DPI**	**Z3**	**Z2**	**Z1**	**PT**	**DPI**	**Z3**	**Z2**	**Z1**	**PT**
1	8.8	13.4	3.2	7	1	140.8	214.4	51.2	112	1	1.4	2.4	0.8	2.4
2	8.7	11.3	2.7	1.4	2	139.2	180.8	43.2	22.4	2	1.4	1.4	1.1	0.4
3	8.8	11.3	2.5	1.7	3	140.8	180.8	40	27.2	3	1.8	1.6	0.8	0.9
4	7.9	10.8	1	4.6	4	126.4	172.8	16	73.6	4	1.0	1.8	1.1	1.7
5	4.2	7.7	3.1	2.8	5	67.2	123.2	49.6	44.8	5	1.8	1.2	0.0	0.0
6	8.1	4.6	2.6	2.2	6	129.6	73.6	41.6	35.2	6	0.0	0.0	0.0	0.0
7	0.9	3.9	2.1	1.8	7	14.4	62.4	33.6	28.8	7	1.7	1.4	0.0	0.0
10	1	4.5	1.1	1.2	10	16	72	17.6	19.2	10	1.5	2.8	0.9	1.1
20	4.5	8.5	1.4	1.1	20	72	136	22.4	17.6	20	1.1	1.6	1.1	0.0
30	4.8	11.1	3.4	1.3	30	76.8	177.6	54.4	20.8	30	1.5	1.3	1.3	0.0
NC	3	4	1.7	1.3	NC	48	64	27.2	20.8	NC	0	0	0	0

Days post-infection (DPI); periportal (Z1), midzone (Z2), central vein (Z3) areas and the portal tract (PT).

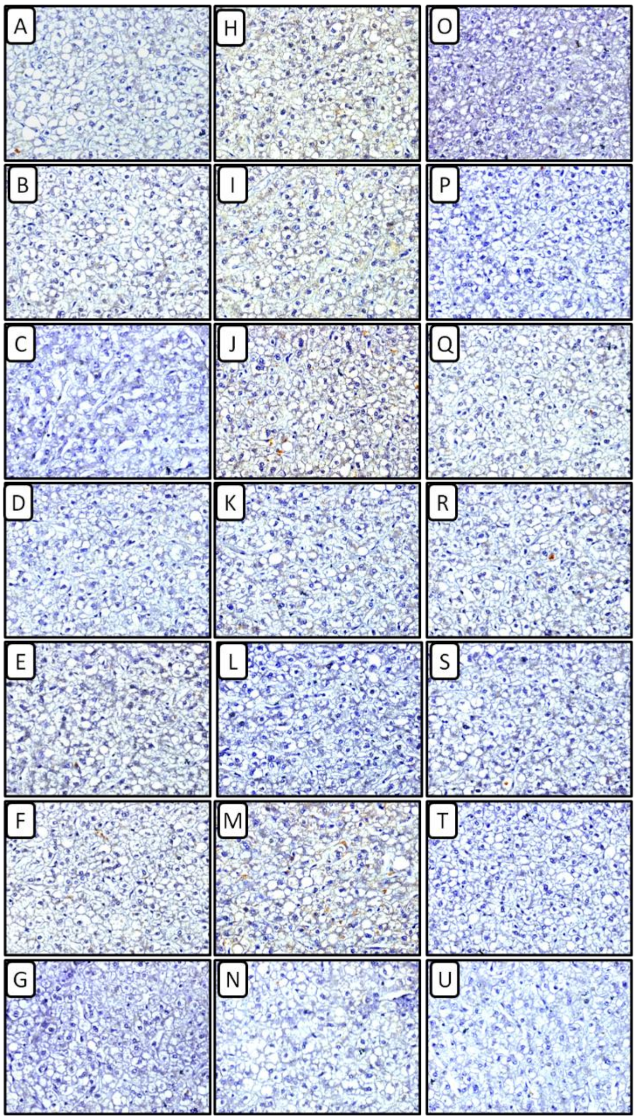

Figure S1. Immunohistochemistry for the various markers in the hepatic parenchyma of the negative control (*Saimiri spp.*) for (A) S100; (B) CD57; (C) CD11b; (D) CD4; (E) CD20; (F) Lysozyme; (G) INOS; (H) Caspase 3; (I) MLKL; (J) IL-4; (K) IL-10; (L) IL-35; (M) TGF-β; (N) IFN-γ; (O) IFN-β; (P) TNF-α; (Q) IL-8; (R) IL-17; (S) ICAM-1; (T) VCAM-1; (U) VLA-4.

Table S3. Immunohistochemistry for the markers in the hepatic parenchyma of the negative control (Saimiri spp).

	**Z3**	**Z2**	**Z1**	**PT**
S100	24	64	3,2	24
CD11B	16	22,4	100,8	17,6
CD57	4,8	12,8	6,4	3,2
CD4	12,8	19,2	4,8	4,8
CD20	9,6	6,4	3,2	4,8
VCAM-1	20,8	30,4	12,8	28,8
ICAM-1	17,6	25,6	19,2	27,2
VLA-4	14,4	20,8	14,4	25,6
Caspase 3	20,8	48	17,6	11,2
MLKL	4,8	6,4	1,6	0
INOS	32	64	32	19,2
Lysozyma	24	27,2	17,6	9,6
IFN-γ	43,2	49,6	12,8	19,2
INF-β	36,8	44,8	17,6	30,4
INF-α	49,6	67,2	17,6	30,4
Il-8	67,2	84,8	20,8	38,4
IL-17	28,8	49,6	27,2	25,6
IL-4	48	65,6	17,6	33,6
IL-10	33,6	57,6	24	49,6
IL-35	33,6	52,8	24	35,2
TGF-β	48	64	27,2	20,8
